# Observations upon a Disease of the Stomach, in Which a Well-Defined Perforation Takes Place in the Tunics of That Organ, without Any Softening of Their Structure. Illustrated by Cases

**Published:** 1828-10

**Authors:** C. H. Ebermaier


### DISEASE OF THE STOMACH.

*Observations upon a Disease of the Stomach, in which a well-defined Perforation takes place in the Tunics of that Organ, without any Softening of their Structure. Illustrated by Cases.* By Dr. C. H. Ebermaier.*

Notwithstanding the attention of the profession has been directed to this subject by many able practitioners, no satisfactory information has yet been recorded which connects the appearances detected upon examination after death with the symptoms which existed during the life of the patients. The cases hitherto collected, in which these circumscribed perforations have been found, with smooth and regular edges, and without any alteration in the structure of the surrounding parts, have been generally in direct contradiction to the opinions formed from the symptoms complained of. In 1803, Gerard endeavoured to deduce some general opinions from the facts which had been stated respecting perforations of the stomach, and to explain the symptoms which took place during life. Chaussier has also described an appearance sometimes detected in the stomach, which might be referred either to the action of poison, or to some external injury, or to disease of the stomach. His work relates principally to perforations produced by softening of the stomach. Since the moderns have become more familiar with this softened state of the stomach and intestines, cases of perforation and spontaneous rupture have been frequently observed and recorded. The disease upon which these appearances depend must consequently be common. The subject of perforations in the stomach with smooth edges has, however, been much neglected.

Case I.—A woman, twenty-two years of age, of robust form, sought assistance for ophthalmia, under which she had laboured for several weeks. This affection was speedily subdued. Dr. E. was then informed that for many years the patient had suffered, almost constantly, from a train of symptoms which would hardly have been suspected from her general appearance of good health. The only mark of ill health was a slight paleness of the face. At the age of eighteen she began to menstruate, and, after having continued regular for about a year, the menses ceased, without any evident cause. For some months she continued in good health. At the end of this period, the digestive functions were much disturbed: her stomach ceased to bear her accustomed food; even the lightest aliment produced considerable pain in the stomach, acid eructations, and pains in the precordia. These

* Journal Complementaire, Juillet 1828.

symptoms gradually increased both in duration and severity, and frequently appeared suddenly after the patient had eaten. Vomitings soon took place some hours after food had been taken; half-digested aliment, mixed with mucus, was thrown from the stomach. The symptoms were not, however, relieved by the stomach being thus freed from its contents. At length the vomiting became almost constant, even after the mildest food, but it was not so violent. The symptoms were not yet, however, so severe as to confine the patient to bed, or to prevent her from following her ordinary occupations, excepting occasionally for a few hours. The nutrition of the body did not appear to be much diminished. So far from this being the case, there were intervals of some months in which the patient enjoyed comparative ease, and during which the spasms of the stomach were so much diminished as to lead to the hope of radically curing a disease which had been considered more distressing than dangerous.

During the first two years, a great variety of means were had recourse to, without any avail. The whole tribe of antispasmodics and emmenagogues were exhausted in vain. The menstrual discharge did not appear. The imperfect digestion, the vomiting, the dull pain in the region of the præcordia, and occasional attacks of fever, continued without diminution. The patient consequently lost all confidence in the power of medicine, and resolved to trust to the efforts of nature alone; which she did during one year.

It was presumed that the derangement of the stomach was produced by the total suppression of the menstrual discharge.

Having again submitted herself to the direction of her physician, she was bled in the foot; a mixture of cream of tartar, sulphur, and chamomile infusion was taken; the feet were frequently immersed in warm water; and the lower part of the abdomen was rubbed with stimulating liniments.

For several months Dr. Ebermaier entirely lost sight of her. He was informed that she was relieved for a short time by the above treatment, but that all the symptoms then returned with their original severity. She was still able to perform her domestic duties, but was incapable of working in the field, on account of the pain she experienced in bending her body. Pressure did not increase the pain she complained of in the epigastrium. She every day carried milk and vegetables the distance of a mile, without inconvenience. The menses had not appeared. Frequently and irregularly spontaneous and easy vomiting took place, two or three hours after she had taken food.

At the end of about seven weeks she died suddenly, to the astonishment of Dr. E., who had still viewed her malady with little apprehension.

Until the day of her death she continued lively in spirit, and capable of performing moderate labour. She rose early, took a little bread and coffee, and went into the garden to gather fruit, which she was to carry to market. She was in the act of stooping, when she suddenly screamed out, with great anxiety, "I am dying," and fell apparently expiring in the greatest torture. Her hands and feet became cold; she complained of excessive pain in the belly; the thirst was inextinguishable, and her general restlessness and anxiety very distressing. There was now no disposition to vomit. She died in a short time.

Upon examining the body, a considerable quantity of fluid was found in the abdomen. In the stomach was found a regularly formed hole, on the anterior part, through which the contents had of course escaped into the abdomen, together with the large quantity of water the patient had taken during the last hours of her existence. Around this aperture there was not the slightest appearance of inflammation, redness, suppuration, ulceration, or erosion; nor any organic lesion whatever. The internal margin of the orifice was perfectly smooth, and the surrounding parts as free from any morbid appearance as the external. The hole, in fact, presented the same appearance as one which would be made in a piece of leather with a punch.*

Case II.—A man, fifty years of age, of a sanguine and bilious temperament, had complained, every two or three months for the last five years, of pains in the belly. He died suddenly. On the right anterior surface of the stomach, a hole, about the size of a two-franc piece, with callous edges, was found. In the small intestines were observed several gangrenous spots. It was ascertained that, five years before the commencement of the symptoms under which he had laboured, he had received a severe blow from the pommel of a saddle on the epigastric region.

Case III.—A girl, fifteen years old, had suffered for two or three years from slight pains in the belly. As her sufferings increased, medical assistance was sought for. She was found to have all the symptoms of enteritis; the face was pale and anxious; urine small in quantity, and of a deep colour. Bowels constipated. The patient could assign no cause for the attack. Some years before she had had a similar attack, and since this time she had been occasionally subject to pains in the stomach.—Ten ounces of blood were taken. A clyster with castor-oil was administered, and emollient fomentations applied to the abdomen. She died in half an hour.

*Appearances post-mortem.*—The omentum adhered to the peritoneum, and at different points to the intestines. The abdomen

* It is worthy of remark, that precisely the same description of a perforation found in the stomach, has been given by Mr. Griffiths, in his account of a somewhat similar case. His words are, "the perforation looked much as if it had been made with a punch." Vide London Medical and Physical Journal for April 1825, p. 289.—In the case related by Mr. Griffiths, the patient had suffered from previous illness for two months; she was attacked suddenly, after eating a hearty breakfast, with great pain in the belly, and died in a few hours.—Editors.

contained a good deal of serous fluid, mixed with coagulable lymph. Throughout the small intestines there were traces of inflammation. The large intestines were also slightly inflamed in different parts, and much distended with air. The liver was smaller and paler than usual. The stomach was empty, and inflamed in different spots. Near the cardiac extremity a circular hole was found, of about nine lines in diameter: its edges were smooth and regular. At the opposite side of the stomach there was another perforation, of an oblong form, but not passing entirely through the external membrane. It appeared as if it had been once completely perforated, but that the orifice had subsequently closed.

Case IV.—A robust man was attacked with a fixed pain in the epigastrium, accompanied by so distressing a throbbing that he was twice bled. After his meals, he vomited both solid and liquid food. For a long time he confined himself to a very light diet, but without any benefit. For a considerable period he suffered from attacks of fever, the pain and vomiting still continuing. He was frequently bled. He at length threw up a considerable quantity of blood, mingled with pieces of substance, some resembling liver, and others like fragments of the villous coat of the stomach. For about three weeks he went on with occasional variations in the severity of his symptoms, when, after a very severe accession of pain and vomiting, he fell a sacrifice to the disease.

Upon examination, the abdominal viscera were found swimming in a mixture of oil and other liquids which the patient had taken. The stomach was free from adhesions to any of the surrounding parts, and without any traces of inflammation. On the right and anterior part of the small curvature a round hole was perceived, about six or seven lines in diameter. The interior of the stomach was perfectly free from any traces of inflammation. The internal orifice of the perforation was much larger than the external. The edges, examined with the finger, appeared hard, solid, and of a cartilaginous nature.

Case V.—A man, twenty-eight years of age, had been frequently troubled, during his youth, with affections of his stomach, which had been attributed to worms. For many years he enjoyed an apparently good state of health. Without any previous indisposition, he was attacked suddenly one evening with violent pain in the belly, which almost bent him double. He was carried home on a board, and threw up from his stomach some bread and wine which he had taken in the morning. A similar mode of treatment was adopted to that in the above cases, but without effect. He died in a few hours.

*Appearances on dissection.*—The contents of the stomach had escaped into the abdomen. At the small curvature of the stomach, about an inch from the pylorus, a hole was found, about a line and a half in diameter, and rounded as if it had been made with a punch. This hole was surrounded by a red circle. The interior of the stomach, and every other organ, were perfectly healthy.

Case VI.—Desgranges attended a woman who for four years had been subject to pains in the stomach, from the severity of which she at length died. She never vomited. A similar aperture was found in the stomach to that above described. In other respects the stomach was perfectly healthy. The intestines were slightly inflamed.

Case VII.—A man had been subject for a considerable time to pains in the stomach. He had sometimes long intervals of ease. He gradually emaciated. Vomiting took place; and, after great and tedious suffering, he died. The pylorus was found in a scirrhous state. Two apertures were seen in the stomach, one an inch in diameter, the other much smaller. There was no appearance of inflammation in any part.*

For many reasons, this communication of Dr. Ebermaier's demands our strictest attention. In the first place, it is of course desirable that all similar cases should be recorded, that we may be enabled, if possible, to establish a diagnosis of so formidable a malady, the symptoms of which, in many cases, unfortunately so much resemble a violent attack of spasm, that the practitioner may easily be led to adopt a mode of treatment which cannot but be prejudicial. The sudden and violent manner, also, in which the patient is not unfrequently attacked and destroyed, may excite suspicions that some poisonous article has been administered; and the most lamentable consequences may ensue, if the medical attendant is not aware of the frequent occurrence of such cases. A case in point occurred in France in 1818. A woman was attacked with violent pains in the stomach, and general symptoms of illness, after having walked some distance on a very sultry day. She had taken no refreshment whatever until her return home, when she partook of a light meal with her husband and some friends. From the commencement of the attack, she was tormented with raging thirst; she had frequent stools, accompanied with great pain in the bowels. She did not vomit. Upon examination post mortem, the stomach was found to be in a state of inflammation, and there was every appearance of some violent caustic having been applied. In some parts the coats of the stomach were entirely destroyed. The pyloric portion was of a deep

* For much additional information upon this very interesting, and as yet obscure subject, our readers may consult the Dictionnaire des Sciences Med. tome xlvi. p. 314, art. Perforation, illustrated by plates.

brown colour, and contracted. After this examination, Dr. R. declared it to be his opinion that poison had been administered. The same opinion was also given by several physicians and surgeons, who were consulted. It was determined that the destruction of the stomach must have arisen from some caustic material, *for that no disease could destroy so large a portion of living animal substance.* Chaussier was fortunately called upon for his opinion: he very properly deprecated the rash and ignorant decision of his brethren, who had made no attempt to prove the presence of poisonous matter. He stated that the same appearances, and the same sudden accession of symptoms, frequently occurred from internal disease. The husband of the woman was consequently acquitted. There was not the slightest grounds for suspicion in this case, excepting the manner in which the woman had been attacked, and the destruction of the stomach which was detected upon examination after death.

We not unfrequently find a partial, or even general, destruction of the stomach, in young children. The symptoms that exist during life in these cases sometimes clearly indicate disease of the stomach and bowels; but almost as frequently they are very obscure, and do not point out any derangement of the digestive organs. In these instances the aperture found in the stomach is very different from that described in the above paper. The edges are ragged, and the whole of the stomach is usually soft and pulpy to the feel. Various parts of the intestines also are generally found to have lost their natural firmness, and are torn if but slightly handled. A very instructive paper has been published by Dr. Gairdner upon this subject in the Transactions of the Med.-Chirurgical Society of Edinburgh, 1824. A paper was also published by Mr. North in our Journal for December 1824, upon the same subject.

By Mr. Hunter, and many other authorities, this destruction of the stomach has been presumed to arise from the action of the gastric juice. We doubt the accuracy of this explanation. Mr. Hunter was led to adopt this opinion from finding these appearances in the stomach where the subject had died suddenly, and where he presumed the gastric juice still retained its activity, and that, consequently, the process of digestion went on after death; while the stomach, being dead, was no longer capable of resisting the powers of that menstruum which itself had effected the digestion of food. But it will be seen, by a reference to the papers we have just mentioned, that most of the children in whom this destruction of the stomach was found had long laboured under very tedious and debilitating diseases. To these cases, then, Mr. Hunter's explanation cannot apply.

Mr. PAXTON observes,* "We have been in the habit of examining a great number of animals at different periods after death, and most of them carnivorous, whose gastric secretion is more active than that of the human stomach in dissolving animal matter; yet in these we never could find any erosion of the coats of the stomach, which must have been the case if it were possible for the gastric juice to have such effects. We consider the stomach, therefore, to be equally insensible to its presence in life or in death."

In a case which was published in the Medical Repository for August 1815, about half the cardiac portion of the stomach was found to be completely destroyed. The preparation was presented to Mr. BROOKES. The child had long been in a very feeble state of health, and at last died in a complete state of marasmus. In such a case there could be no reason for presuming that the gastric juice possessed any unusual activity: on the contrary, its powers must have been weakened in proportion to the debility of the viscus which secreted it.—EDITORS.

* PALEY'S Natural Theology, illustrated by Plates and explanatory Notes, by JAMES PAXTON.

